# Physical Activity and Depression in Adolescents: Evidence from China Family Panel Studies

**DOI:** 10.3390/bs12030071

**Published:** 2022-03-08

**Authors:** Jiafeng Gu

**Affiliations:** Institute of Social Science Survey, Peking University, Beijing 100871, China; isssgujf@pku.edu.cn

**Keywords:** physical activity, adolescents, depression, housework, logistic regression

## Abstract

Depression in adolescents is a major public health disorder. The relationship between physical activity and risk of depression in adolescents was examined using three waves of data from the China Family Panel Studies in 2020. The risk of depression was significantly higher among adolescents who reported lower frequency and shorter duration of physical exercise than those who reported physical exercise more frequently and for a longer duration. The risk of depression was significantly higher among adolescents who reported intense physical exercise than those who reported little or no intense physical exercise. The amount of time spent on housework by adolescents is inversely associated with depression. These results provide somewhat stronger evidence for an activity–depression link than previous studies and suggest a differential role for different types of physical activity, such as exercise and housework. The overall model predicting depression in adolescents (LR chi-squared = 95.974, *p* < 0.001, Nagelkerke R-square = 0.183) was statistically significant. To effectively control depression in adolescents, the government, schools and parents need to act together to guide adolescents towards participation in appropriate physical activities. The appropriate level of physical activity is for adolescents to experience breathing, rapid heartbeat, and slight perspiration.

## 1. Introduction

Depression is a mood disorder with the highest incidence of psychological problems in adolescents, with significant and persistent depression as the main clinical feature [1]. Adolescents, as a high-level reserve talent echelon in society, have received active attention from society for their mental health issues. In recent years, the depression rate among adolescents has been on the rise, and “silent killers” are targeting adolescents [2]. The prevention and treatment of depression in adolescents is a matter of urgency. Adolescence is a critical stage for developing healthy habits [3]. In 2021, the Chinese Ministry of Education began to include depression screening in the context of students’ health check-ups. However, more work is needed to truly keep “silent killers” away from adolescents.

Many studies have demonstrated the positive impact of physical activity on adolescent mental health [4,5]. However, the problem of insufficient exercise among adolescents is becoming increasingly severe. Globally, in 2016, 81% of school-going adolescents aged 11–17 did not meet current recommendations of at least one hour of physical activity per day [6]. The Chinese National Nutrition and Health Survey in 2002 revealed that 32% of urban adolescents reported that they seldom exercised [7]. In 2020, the depression rate among Chinese adolescents was 24.6% [8]. How to encourage adolescents to participate in physical exercise and effectively use exercise methods to intervene in adolescent depression has become one of the main objectives of adolescent education.

The effect of physical activity on depression remains controversial in the academic community. Many studies have shown that physical exercise is an effective way to alleviate depression [9,10,11]. However, some studies have also found that the effects of exercise on the relief of depression are “moderate at best” or statistically insignificant [12,13,14]. This discrepancy suggests the need for further research and testing of antidepressants for physical activity. In addition, there are different types of physical activities. In general, physical activities can be divided into exercise activities and non-exercise activities [15]. Exercise activities refer to those structured physical activities that require relatively high energy consumption over a period of time, such as swimming, running, playing, and fitness [16]. Non-exercise activities refer to those habitual daily activities, such as going home and climbing stairs, doing housework, taking out the trash, going out to pick up express delivery, etc. [17]. Existing studies have mainly focused on the effects of physical exercise on depression in adolescents, whereas studies on the effects of non-physical exercise are relatively lacking. In addition, the Chinese government is also implementing the “double reduction” policy, which aims to reduce the burden of excessive homework and off-campus training for students in compulsory education, and to prevent depression among teenagers. However, there is no systematic evaluation of the effect of the “double reduction” policy on depression prevention in adolescents.

Therefore, the purpose of this study was to determine the efficacy of physical activity, including exercise activities and housework, on depression in adolescents by conducting a logistic analysis of the data available from China Family Panel Studies in 2020. The main contributions of this study are as follows: first, testing the effectiveness of the activity–depression link in a Chinese context can enrich this field; second, reconceptualizing the influence of housework on adolescent depression can help to reveal the influence of non-exercise activities more comprehensively; third, discussion of the current “double reduction” policy implemented in China will help to improve the practical insight of research on the activity–depression link.

The rest of the paper is organized as follows: Section 2 presents the literature review and hypothesis development; Section 3 presents the data, samples, and methods; Section 4 presents the results; Section 5 discusses the results; Section 6 concludes the paper.

## 2. Literature Review and Hypothesis Development

Appropriate physical exercise is generally considered to be beneficial for relieving depression in adolescents [9]. Physical exercise is effective in relieving symptoms of depression [10]. Physical exercise may be effective in improving depressive status, hormonal response to stress, and physiological fitness in adolescent females with depressive symptoms [18]. Increased physical activity plays an important role in promoting health and preventing lifestyle-related psychological disorders in adolescents [5]. A randomized controlled trial study has shown that exercise intervention can reduce depressive symptoms [11]. One of the values of physical exercise in reducing adolescent depression is that exercise fits within the natural ecology of adolescent activities, whereas psychotherapy and psychotropic medications do not [19]. In addition, different forms of exercise have different effects on adolescent depression. Aerobic exercise can effectively relieve depression in adolescents, but anaerobic exercise and leisure have no significant effect on reducing depression in adolescents [20]. Therefore, research on the effect of physical activity on adolescent depression also requires dialectical analysis.

Physiology supports the role of physical exercise in reducing depression in adolescents. The production of β-endorphins may be increased by regular physical exercise [21]. With the stimulation of endorphins, adolescents are in a relaxed and happy state of mind and body [22]. Endorphins are also known as “happiness hormones” or “youth hormones”, which means that they help adolescents stay happy and older people stay young [23]. Appropriate intensity of physical exercise can speed up blood circulation to the brain and various organs of the body, promote metabolism, and relax muscles to ease the mind [24]. Physical exercise has a regulatory effect on the secretion of neurotransmitters in the human body. Typical neurotransmitters such as adrenal cortex, serotonin, and dopamine can promote an individual’s positive emotional experience of excitement and pleasure and improve the ability to resist depression [25]. Neurotransmitters need a certain concentration to be effective. High-frequency, long-term physical exercise ensures the accumulation of neurotransmitters in the brain and lays the foundation for improving mood and relieving depression in adolescents [26]. The evidence from a meta-analytical study shows that a positive correlation exists between physical activity levels and reduced levels of depression [27].

Therefore, the following hypotheses are proposed:

**Hypothesis** **1** **(H1).** *Frequency of physical exercise is negatively correlated with depression in adolescents*.

**Hypothesis** **2** **(H2).** *Physical exercise duration is negatively correlated with depression in adolescents.*

Although studies consistently demonstrate that physical exercise can enhance emotional well-being and help treat depression, overly strenuous exercise can also have side effects that can make people more depressed. That is, the relationship between physical exercise intensity and depression in adolescents is likely to be nonlinear. Excessive exercise can lead to mental fatigue, which correlates with depression [28]. Excessive physical activity may lead to overtraining and produce depression-like psychological symptoms [29]. Excessive exercise can lead to exercise-induced anemia, which in turn can lead to depression [30]. Excessive physical activity increases the chances of respiratory infections, injuries, and chronic pain [31]. Considering the possible negative effects of excessive exercising on depression, numerous studies have investigated the appropriate intensity of exercise for children and adolescents [5,32,33]. These studies suggest that there may also be a positive correlation between physical exercise and depression if the physical exercise is too intense [34]. It has also been shown that moderate-to-vigorous physical exercise is beneficial and may improve fatigue and depressive symptoms in young people with pediatric-onset multiple sclerosis and other demyelinating disorders [35]. However, the academic community generally agrees with this judgment: overdone is worse than undone. Therefore, the following hypothesis is proposed:

**Hypothesis** **3** **(H3).** *High-intensity physical exercise is positively correlated with depression in adolescents.*

Housework refers to a kind of unpaid work that family members must perform in their daily lives, including: washing and cooking, buying daily necessities, cleaning and hygiene, caring for the elderly or sick, etc. A study based on 83 participants aged 18–28 showed that daily housework could improve well-being [15]. However, more research suggests that housework may exacerbate depression. A study based on 2341 women between the ages of 60 and 79 years from 15 British towns showed that women who reported depression were less likely to engage in heavy housework [36]. A study of 73 Japanese women found that housework was associated with the presence of depression [37]. In Korea, the more time adolescents spent doing housework alone, the more severe the symptoms of depression [38]. In the United States, a study of adolescents aged 8–18 years from 201 European American families showed that time spent on housework was associated with more depressive symptoms [39]. Housework is sometimes seen as a burden, and men are reluctant to share the burden of household chores [40]. Similarly, for adolescents, housework is often seen as a burden that they are unwillingness to take on. In the face of children who are unwilling to cooperate, some parents resort to simple and rude methods such as forcing their children to do housework, which leads to instant tension in the parent–child relationship and even escalates parent–child conflict. Under these conditions, it is obvious that doing housework can make adolescents more depressed. Therefore, the following hypothesis is proposed:

**Hypothesis** **4** **(H4).** *Housework duration is positively correlated with depression in adolescents.*

**Figure 1:**
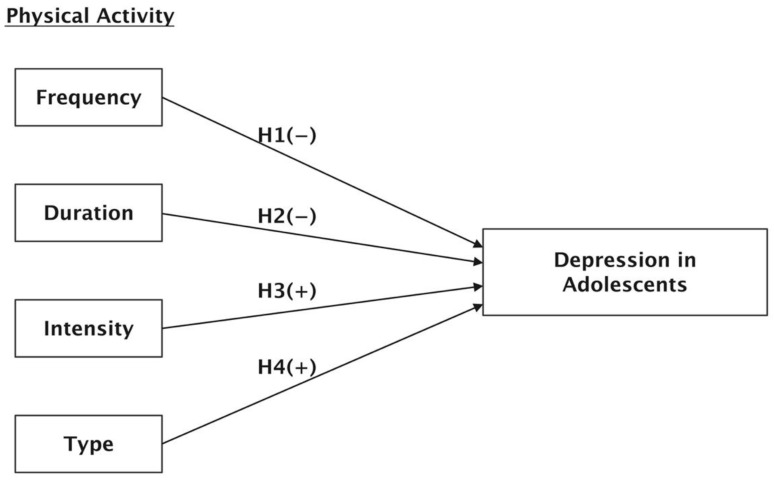
Theoretical framework and related hypotheses.

Generally, physical activity has four dimensions, namely frequency, duration, intensity, and type. Based on these four dimensions, the theoretical framework of this study was formed and the relevant hypotheses were established. The theoretical framework and related hypotheses are summarized in Figure 1.

## 3. Methods

### 3.1. Sample

Data for this study were obtained from the China Family Panel Studies (CFPS), a comprehensive survey based on individuals, families, and communities conducted by the Institute of Social Science Survey (ISSS) at Peking University, China. CFPS data are widely used in research in the field of health in China [41,42,43,44]. The CFPS sample consists of 25 provinces, municipalities, and autonomous regions in mainland China except for Xinjiang, Tibet, Qinghai, Inner Mongolia, Ningxia, and Hainan. The population of this sample represents 95% of the population of mainland China. During the particular historical period when COVID-19 was rampant, the 2020 CFPS survey was conducted using computer-assisted telephone interviewing (CATI) or online interviewing via computer-assisted web interviewing (CAWI). Individual response questionnaires from the 2020 follow-up study were used for this study. The dataset included 3360 adolescents between the ages of 10 and 19, with a total of 28,590 people interviewed. Subsequently, adolescent samples with missing values were removed. Specific sample exclusion criteria included: those with missing socio-demographic information required for the study; those with missing items in the Depression Scale; and those unable to obtain depressive symptoms. The final adolescent sample (n = 896) provided the data for this study.

### 3.2. Measures

#### 3.2.1. Dependent Variables of Depression

The Center for Epidemiologic Studies Depression Scale (CES-D) was used by CFPS2020 to measure an individual’s level of depression. This set of scales comes in a variety of forms. In CFPS2012, a 20-question CESD20 was used. However, feedback from the field survey showed that the scale used in the CFPS personal questionnaire had too many questions. Beginning in 2016, a simplified model of the CES-D scale was used, with the number of questions reduced from 20 to 8, namely the CESD8. Meanwhile, in order to effectively compare depression scores between different rounds, we randomly selected 1/5th of the sample of respondents to continue using the CESD20 and the remaining 4/5th to continue using the CESD8. Based on this design, data processors at a later stage equalized the scores of the two sets of items, using the equipercentile equating method to generate a comparable score—CESD20sc (the constructed CESD20 total score). This score maintains the CESD20 scoring interval and is also comparable to the CESD20 scale score in CFPS2012. The alpha coefficient of this scale is 0.794, which has good reliability. Currently, this scale is widely used to screen for depressive symptoms with good reliability and validity, and has been proven to be applicable to Chinese residents [45]. The higher the score, the more severe the depressive symptoms. According to the relevant research, a score above 27 points can be considered as “having depressive symptoms”. The operational definition of the depression variable is as follows: a score above 27 points is defined as 1, while others are defined as 0.

#### 3.2.2. Physical Activity

Regarding the frequency of physical exercise, respondents were asked, “How often do you participate in physical activity?” Responses were categorized into the following six levels: less than 1 time per month on average; more than 1 time per month on average, but less than 1 time per week; 1–2 times per week on average; 3–4 times per week on average; 5 or more times per week on average; 1 time a day. The above six responses were coded from 1 to 6 to obtain the variable of physical exercise frequency. Thus, this variable is a continuous variable, with larger values indicating more frequent exercise. Regarding the duration of physical exercise, respondents were asked, “In the past 12 months, how many minutes did you exercise at a time?” As such, this variable is also a continuous variable. Regarding exercise intensity, respondents were asked, “How do you feel when you regularly participate in physical exercise?” The answers were: little change in breathing and heartbeat compared with when not exercising; rapid breathing, faster heartbeat, slight sweating; shortness of breath, markedly faster heartbeat, and more sweating. According to the answers, the exercise intensity can be divided into three categories, namely, non-intense, slightly intense and intense. Therefore, this variable is a categorical variable. Regarding the time spent on housework, respondents were asked, “How many hours do you spend on housework every day?” Therefore, this variable is also a continuous variable.

#### 3.2.3. Control Variables

The control variables were gender, happiness, age, health status, changes in health, education level, type of residence, family type, and city type. Gender is a binary variable: males are defined as 1 and females as 0. Females are set as the baseline group. Regarding personal happiness, respondents are asked “How happy are you (points)?”, and the answer is a minimum score of 0 and a maximum score of 10. It is a continuous variable. In terms of age, 95 adolescents were 10 years old, 97 were 11 years old, 80 were 12 years old, 93 were 13 years old, 89 were 14 years old, 95 were 15 years old, 89 were 16 years old, 72 were 17 years old, 98 were 18 years old, and 88 were 19 years old. Regarding personal health status, respondents were asked, “How do you consider your health to be?”, and the answers were: extremely healthy, very healthy, relatively healthy, average and unhealthy. Health status is categorized into four levels. Regarding changes in personal health, respondents were asked, “How do you consider your health compared to a year ago?”, and the answers were: got better, no change or got worse. Change in health status is divided into three categories. Education level was categorized as (0) primary school, (1) junior high school, (2) high school, and (3) college. Type of residence was categorized as (0) rural and (1) urban. Rural was set as the baseline group. Family type was categorized as (0) non-mid-high net worth (MHNW) families and (1) MHNW families. Rural was set as the baseline group. If the family’s investable assets are greater than or equal to RMB 450,000, it is an MHNW family. If they are not, then it is a non-MHNW family. The non-MHNW family was set as the baseline group. City type was categorized as (0) first-tier cities, (1) second-tier cities, (2) third-tier cities, and (3) others. First-tier cities were set as the baseline group.

### 3.3. Statistical Modeling

The binary correlation between categorical explanatory variables and depression in adolescents was described by chi-square analysis. Data were cleared and processed by StataMP 16. Multivariate logistic regression was used to test each hypothesis in the theoretical model.

## 4. Results

Adolescents aged 10 to 19 years are listed in Table 1. In 2020, 26.56% of adolescents were found to have depressed mood or depressive symptoms (n = 238) and 73.44% of adolescents were not found to have depressed mood or depressive symptoms (n = 658). Table 1 is a binary cross-tabulation reflecting that exercise intensity, health status, changes in health, and family type may all be associated with adolescent depression. In contrast, gender, education level, type of residence, and city type were probably not associated with adolescent depression.

Table 2 shows the results of multivariate logistic regression analyses for the four categories of physical activity and control variables. Increasing the frequency of exercise reduced the likelihood of depression in adolescents (OR: 0.892; 95% CI: 0.799–0.955). Thus, Hypothesis 1 is confirmed. Regular participation in physical exercise can help adolescents relieve stress and expand their social circles, thus effectively reducing the probability of depression. The duration of each exercise session was negatively correlated with the probability of depression in adolescents (OR: 0.997; 95% CI: 0.993–1.001). Thus, Hypothesis 2 is confirmed. As long as the intensity of physical exercise is moderate, a longer duration of exercise will allow adolescents to better immerse themselves in the excitement of exercise and forget about possible troubles and worries, thus reducing the likelihood of experiencing depression. Regarding exercise intensity, general exercise intensity did not cause depression in adolescents, but excessive intense exercise may increase the probability of depression in adolescents (OR: 1.723; 95% CI: 1.059–2.804). Thus, Hypothesis 3 is confirmed. In addition to causing possible injuries to adolescents, excessive strenuous exercise may also deplete adolescents’ energy and prevent them from concentrating on their studies, thus making them emotionally depressed. In addition, it was found that doing housework could increase the probability of depression in adolescents (OR: 1.187; 95% CI: 1.018–1.385). Thus, Hypothesis 4 is confirmed. This is a phenomenon worthy of attention. Housework is not only a physical activity that is beneficial to the physical and mental health of adolescents, but it may also become a burden for them. Therefore, it is necessary to dialectically analyze the impact of housework.

The happier adolescents felt, the less likely they were to experience depression (OR: 0.801; 95% CI: 0.731–0.867). The likelihood of depression was not significantly related to the age of the adolescent (OR: 0.801; 95% CI: 0.731–0.867). Regarding health status, the healthier the adolescent, the lower the probability of depression (OR = 0.175; 95% CI: 0.022–1.367/OR = 0.719; 95% CI: 0.647–0.898). Regarding changes in health status, adolescents who felt less healthy were more likely to suffer from depression (OR = 3.065; 95% CI: 1.22–7.698). Regarding education level, adolescents with primary school as the highest level of education were less likely to be depressed (OR = 0.538; 95% CI: 0.308–0.942). Regarding city type, adolescents living in second-tier cities were more likely to be depressed (OR = 2.206; 95% CI: 0.873–5.579). In addition, the results of the study show that adolescent gender, age, type of residence and family type had no significant relationship with depression.

## 5. Discussion

Depression in adolescents is a problem of widespread social concern. Adolescent depression is a psychological disorder that occurs in adolescents with a high incidence worldwide and significantly increases the risk of suicide, which is the second leading cause of death among adolescents [46,47]. Meanwhile, adolescents are also at increased risk of developing depression [48]. This study found that the depression rate among adolescents reached 26.56%. The “Report on the Development of Chinese National Mental Health (2019–2020)”, issued by the Institute of Psychology of the Chinese Academy of Sciences, mentioned that, in 2020, the depression rate among Chinese adolescents was 24.6% [8]. It can be seen that the depression rate among adolescents reported in this study is higher than that study. An important reason for this difference is the different sample composition. In this study, the urban and rural distribution of the youth sample was relatively balanced, with urban youth accounting for 55.45% and rural youth accounting for 44.55%. However, in that study, the urban youth sample accounted for 35.5%, while the rural youth sample accounted for 64.5%. Although there was no significant difference in the level of depression between urban and rural adolescents in this study, it has also been shown that urban adolescents (14.64%) were more depressed than their rural counterparts [49,50]. Overall, almost one in four Chinese adolescents suffer from depression.

This study shows that both the frequency and duration of physical exercise can help reduce depression in adolescents. Effective physical exercise can improve adolescents’ mental health and physical health. This is consistent with the findings of a series of previous studies [9,10,27]. The treatment of depression by physical exercise, especially the interpersonal effects of depression in adolescents, has some effects that cannot be replaced by medications and other therapies. Adolescents who regularly participate in physical exercise are more likely to form good interpersonal relationships with others, and also enhance their social adaptability and good psychological regulation. As can be seen, the results of this study show that physical exercise has a positive effect on reducing depression in adolescents, which is different from previous studies showing that the effects of exercise on depression relief are “moderate at best” or statistically insignificant [12,13,14]. A possible explanation is that the effect of exercise on depression in adolescents may be influenced by some moderator variables that are not clearly defined and identified now. Overall, the effect of physical exercise on reducing depression is definitely predominant. Regular physical exercise can improve the negative emotions of adolescents, thus indirectly influencing and reducing the occurrence of depression.

Although physical exercise is important for the mental health of adolescents, the problem of physical inactivity among adolescents is also prominent. From a global perspective, the daily physical activity status of adolescents in many countries is not optimistic. The proportion of adolescents who are physically active for less than 1 h per day at moderate intensity is as high as 80.3%, and the proportion is higher for girls than for boys [51]. In the United States, less than 20% of adolescents (aged 12–17 years) meet the recommended physical activity guidelines [52]. In China, this issue is also not optimistic, 32% of Chinese adolescents in cities report seldom exercising [7]. One of the biggest problems in the education of today’s adolescents is the rising rate of depression and lack of physical exercise. To solve this problem, a joint effort of the government, schools and parents is needed. There is an urgent need to reflect on the essential issues of adolescent development in educational theory and practice, to fully examine the physical diversity of the human body, and to focus on the diversity of individuals and the individuality of development. At the government level, in July 2021, China introduced a set of “double reduction” rules to ease the burden of excessive homework and off-campus tutoring on young students [53]. The “double reduction” policy means that adolescents will have more discretionary time and therefore more time to participate in physical exercise. For schools, in the education and teaching work of adolescents under the “double reduction” policy, it is not only necessary to impart cultural knowledge to students, but also to increase physical exercise and training, so that they can relieve their learning pressure during exercise. The “double reduction” policy means that adolescents have more time for extracurricular activities. Parents should cultivate the habit of daily physical exercise for their children, thus sharpening their willpower and enabling them to have good mental health.

While strengthening youth exercise, it is also important to pay attention to the appropriate intensity. This study suggests that excessive strenuous physical activity can actually exacerbate depression in adolescents. This is consistent with previous studies [28,30,31]. These studies emphasize moderation of physical exercise. Excessive exercise can lead to poor self-perception, sleep disturbance, and loss of appetite [35]. For adolescents, the appropriateness of exercise intensity can be judged on the basis of the following two criteria: whether the heart rate basically returns to normal 5 to 10 min after exercise; and whether adolescents feel tired the next day. If the answer to the first question is “yes” and the answer to the second question is “no”, then such exercise intensity is appropriate for adolescents and will not lead to depression in adolescents. If the exercise causes shortness of breath, markedly faster heartbeat, and more sweating, then care should be taken to avoid a situation where adolescents feel depressed due to excessive exercise. At present, the appropriate exercise intensity for adolescents is still controversial because the appropriate exercise intensity varies based on factors such as different regions, different ages, and physical fitness [5,32,33]. However, the basic principle of “overdone is worse than undone” is widely accepted. Therefore, it is important to manage the intensity of exercise and ensure moderate intensity while treating adolescent depression by strengthening physical exercise.

Housework is also an important physical activity. This study found a positive relationship between hours of housework and adolescent depression. That is, the longer adolescents engaged in housework, the more likely they were to develop depression. This finding is consistent with that of most previous studies [36,37,40]. Due to the one-child policy in China, for a long period of time, most children have been the only child in the family and are the “little sun” of the family [54]. Therefore, parents will not let their children touch anything other than learning and playing, especially household chores, and will not let their children do them. Due to this pampering, adolescents are prone to the idea of being unwilling to do housework. When adolescents are forced to do chores by their parents, they will complain and feel depressed. This situation is very different from Western society [15]. If parents can make their children realize the significance of doing housework, performing housework is conducive to developing adolescents’ independent living ability, improving their physical coordination ability, and contributing to the physical and mental health of adolescents. For parents, properly guiding their children to do housework can make them realize the responsibility of the family and understand that housework is not the responsibility of one person or the parents alone, but the responsibility of the entire family. In this family atmosphere, children will learn how to have a sense of responsibility and be willing to participate in household chores. If children are unwilling to do housework, forcing them to do it will make them more rebellious, which is not good for their mental health. When children start doing housework, most of them will be rough and careless. At this time, parents should not be very demanding, but should give their children some encouragement. A study in South Korea found that the more time teens spent doing housework with their mothers, the lower their depressive symptoms were, but the more time they spent doing housework alone, the higher their depressive symptoms were [38]. Therefore, it is very important for parents to do housework with their children so that they can effectively prevent depression.

Although this study achieved the valuable results described above, there are some limitations. In adolescent depression, no distinction was made between mild depression, moderate depression, and severe depression. In the future, the degree of depression in adolescents can be distinguished and the factors affecting different degrees of depression can be further explored. The data used in this study are cross-sectional data used to explore correlations. In the future, panel data could be used to test for causality. This study has explored the effect of adolescent physical activity on depression, but has not explored the effect of adolescent physical activity on depression in adulthood, which is also an important direction for future research. In addition, this study did not explore the effects of sleep time, study time, and social interaction time on depression in adolescents. In the future, these need to be explored and tested. There may also be mediating variables in the effect of exercise on depression in adolescents, which also need to be examined in the next step.

## 6. Conclusions

High rates of diagnosable depression in adolescents present challenging clinical and research issues. The results of this study show that the frequency and duration of physical exercise participation are significantly and negatively associated with depression in adolescents. This illustrates the importance of youth participation in physical exercise. However, excessive exercise increases the likelihood of depression in adolescents. Therefore, while encouraging adolescents to participate in physical exercise, it is necessary to guide them to participate in exercises of appropriate intensity according to their own condition and physical fitness. The study also shows that the length of time adolescents participated in household chores was significantly and positively associated with depression. Therefore, parents should pay attention to the way they guide their children to do housework.

## Figures and Tables

**Table 1:** Distribution of general characteristics.

	Number of Participants (n = 11,547)		Drinking		$x^{2}(p)$
	Classification	N (%)	Yes N (%)	No N (%)	
Exercise intensity	Non-intense	119 (13.28)	50 (16.56)	69 (11.62)	9.473 *** (0.009)
	A little intense	460 (51.34)	164 (54.3)	296 (49.83)	
	Intense	317 (35.38)	88 (29.14)	229 (38.55)	
Gender	Female	385 (42.97)	131 (43.38)	254 (42.97)	0.031 (0.86)
	Male	511 (57.03)	171 (56.62)	340 (57.24)	
Health status	Average or unhealthy	21 (2.34)	1 (0.33)	20 (3.37)	29.147 *** (0.000)
	Relatively healthy	272 (30.36)	74 (24.5)	198 (33.33)	
	Very healthy	295 (32.92)	91 (30.13)	204 (34.34)	
	Great healthy	308 (34.38)	136 (45.03)	172 (28.96)	
Changes in health	No change	469 (51.34)	169 (55.96)	300 (50.51)	15.387 *** (0.000)
	Got better	369 (41.18)	127 (42.05)	242 (40.74)	
	Got worse	58 (6.47)	6 (1.99)	52 (8.75)	
Education	Primary school	248 (27.68)	76 (25.17)	172 (28.96)	2.592 (0.459)
	Junior high school	280 (31.25)	103 (34.11)	177 (29.8)	
	High School	241 (26.9)	78 (25.83)	163 (27.44)	
	College	127 (14.17)	45 (14.9)	82 (13.8)	
Type of residence	Rural	468 (55.45)	149 (52.65)	319 (55.45)	1.351 (0.245)
	Urban	376 (44.55)	134 (47.35)	242 (43.14)	
Family type	non-MHNW families	781 (87.17)	255 (84.44)	526 (88.55)	3.031 * (0.082)
	MHNW families	115 (12.83)	47 (15.56)	68 (11.45)	
City type	first-tier cities	35 (3.91)	18 (5.96)	17 (2.86)	5.14 (0.162)
	Second-tier cities	56 (6.25)	18 (5.96)	38 (6.4)	
	Third-tier cities	30 (3.35)	10 (3.31)	20 (3.37)	
	Others	775 (86.5)	256 (84.77)	519 (87.37)	
Depression	Yes	238 (26.56)			
	No	658 (73.44)			

Note. ***, *p* < 0.01; *, *p* < 0.1.

**Table 2:** Multivariate logistic regression analysis.

Characteristics	Odds Ratio	Drink_Y_18 95% CI	*p* Value
Frequency of exercise	0.892 **	0.799–0.995	0.04
Exercise duration	0.997 *	0.993–1.001	0.093
Exercise intensity (Re: non- intense)			
A little intense	1.208	0.765–1.908	0.417
Intense	1.723 **	1.059–2.804	0.028
Housework time	1.187 **	1.018–1.385	0.029
Gender (Re: female)			
Male	1.022	0.738–1.417	0.894
Happiness	0.801 ***	0.731–0.876	0.001
Age	1.062	0.922–1.224	0.83
Health status (Re: average or unhealthy)			
Relatively healthy	0.18	0.023–1.406	0.102
Very healthy	0.175 *	0.022–1.367	0.096
Great healthy	0.114 **	0.015–0.886	0.038
Changes in health (Re: no change)			
Got better	1.066	0.772–1.47	0.698
Got worse	3.065 **	1.22–7.698	0.017
Education (Re: illiterate/semi-literate)			
Primary school	0.538 **	0.308–0.942	0.03
Junior high school	0.521	0.211–1.285	0.157
High School	0.406	0.125–1.322	0.134
Type of residence (Re: rural)			
Urban	0.923	0.654–1.304	0.651
Family type (Re: non-MHNW families)			
MHNW families	0.805	0.501–1.295	0.372
City type (Re: first-tier cities)			
Second-tier cities	2.206 *	0.873–5.579	0.095
Third-tier cities	2.074	0.716–6.007	0.179
Others	1.812	0.842–3.901	0.129
Constant	33.794 **	1.874–609.313	0.017
LR chi-squared	95.974 (0.000)		
−2Log likelihood	980.758		
Cox and Snell R square	0.107		
MacFadden square	0.089		
Nagelkerke square	0.183		

Note. ***, *p* < 0.01; **, *p* < 0.05; *, *p* < 0.1.

## Data Availability

The data for this research can be applied and downloaded from the Peking University Open Research Data Platform website (https://opendata.pku.edu.cn/dataverse/CFPS, accessed on 25 June 2021).
